# Prevalence and risk factors of peri‐implant diseases at patient‐level: A cross‐sectional study in Syria

**DOI:** 10.1002/cre2.792

**Published:** 2023-09-28

**Authors:** Asmaa A. A. Aljalloud, Suleiman Dayoub, Yasser Alsayed Tolibah

**Affiliations:** ^1^ Department of Periodontology Faculty of Dentistry, Damascus University Damascus Syria; ^2^ Department of Pediatric Dentistry Faculty of Dentistry, Damascus University Damascus Syria

**Keywords:** peri‐implant diseases, prevalence, risk factors

## Abstract

**Objectives:**

This research aims to assess the prevalence of peri‐implant diseases and to analyze variables of the probable risk at the patient level associated with the occurrence of peri‐implant diseases in Syrian patients.

**Materials and Methods:**

A cross‐sectional study has been carried out on 142 patients with 380 dental implants placed between 2015 and 2021. Patients were invited by phone to return to Damascus University's Periodontology Department for clinical and radiological examination. A descriptive statistical analysis was implemented for the prevalence of peri‐implant diseases at the level of the patients. Also, the peri‐implant diseases' factors of risk were determined by the multivariate analytical model.

**Results:**

The prevalence rate of peri‐implant mucositis and peri‐implantitis in patients was 58.5% and 25.4%, respectively. Peri‐implant disease is associated with multivariate risk indices, gender female (peri‐implant mucositis [OR = 0.269; 95% CI: 0.131−0.552] and peri‐implantitis [OR = 0.561; 95% CI: 0.561−0.216]), diabetes (peri‐implant mucositis [OR = 3.4; 95% CI: 1.73−12.73]), periodontitis (peri‐implant mucositis [OR = 2.409; 95% CI: 1.760−2.613], peri‐implantitis [OR = 10.445; 95% CI: 4.097−26.629]).

**Conclusions:**

Peri‐implant diseases are common in the Syrian community. Several patient‐level variables (gender female, diabetes, and periodontitis) are associated with peri‐implant disease.

## 1 INTRODUCTION

The use of dental implants currently increases 14% yearly and is expected to reach 23% in 2026 (Moraschini et al., 2015). However, this increase in the use of dental implants has resulted in problems and complications such as peri‐implant diseases (Sanz et al., 2019).

Creating a soft tissue barrier around the dental implant at the point it enters the oral cavity is an important step in the implant's functional performance and ensures the esthetic integration of prosthetic restorations. Maintaining this seal in health condition is essential for the long‐term functioning and prognosis of the implant. The essential goal of peri‐implant soft tissue sealing is to protect the osseointegration between the implant and bone (Roccuzzo & Roccuzzo, 2023).

Dental implants could be affected by two types of diseases. The first is peri‐implant mucositis is an inflammation that only affects the soft tissue surrounding the implants, and the second is peri‐implantitis which affects both the soft and hard tissues and is characterized by a gradual loss of the alveolar bone (Berglundh et al., 2018).

A causal relationship between bacterial biofilm accumulation around dental implants and the progression of the inflammatory response has been demonstrated (Heitz‐Mayfield & Salvi, 2018). Moreover, it has been indicated that peri‐implant diseases particularly peri‐implantitis tend to progress quicker than periodontitis, and may take place during the first year of the functional loading of implants (Derks et al., 2016a).

Due to the high diffusion of peri‐implant diseases, researchers have become more interested in investigating such diseases (Heitz‐Mayfield & Salvi, 2018).

Many studies have reported different rates of diffusion for the peri‐implant mucositis (23.9%−88.0%) at the patient's level and (9.7%−81.0%) at the implants' level and the peri‐implantitis (8.9%−45%) and (4.8%−23.0%) at the patient's level and the implants' level, respectively. The diffusion of the peri‐implant mucositis depends generally on the studied population because it is diagnosed by the BOP only while each peri‐implantitis report includes various diagnostic standards (Derks et al., 2016a).

To improve the understanding, the peri‐implant diseases and to be able to implement effective prevention, it is important to comprehend their epidemiology in various geographic areas. Therefore, it is suggested that epidemiological studies and clinical and radiological investigations are inevitable to study the prevalence and the factors of risk that are associated with occurrence of the peri‐implant diseases (Zitzmann & Berglundh, 2008).

Since peri‐implant diseases are opportunistic infections, it is logical to presume that suboptimal conditions, local and/or systemic, may support the progression of peri‐implantitis. Some of these conditions may be the result of poor planning and/or incorrect implementation of any step in the entire course of treatment (Roccuzzo, Imber, et al., 2023).

Knowing the risk factors for peri‐implant disease is very important to remain authentic, as several patient‐related indicators have been reported, including smoking, poor oral hygiene, periodontitis, and no periodic follow‐up (Zitzmann & Berglundh, 2008).

Studies have recognized several risk indices for peri‐implant disease, such as patient‐related factors, and environmental factors, such as periodontitis and smoking, and implant design. A recent consensus report specified a history of periodontitis, poor plaque control, and no routine maintenance therapy as risk indices for peri‐implant disease (Zhao et al., 2022).

In some studies, other indicators, such as systemic disease including diabetes, periodontal status, and prosthetic design, have also been suggested as risk factors, but many studies are not based on a multi‐level analysis that includes all potential factors (Wada et al., 2021).

Patients with periodontitis and non‐acquiescent with supportive periodontal care (SPC) have a higher risk of biological complications and implant failure (Roccuzzo et al., 2022),

In particular, those with severe periodontal disease were 2.5 times more likely to have peri‐implantitis (Arunyanak et al., 2019).

Prevention of incidents of peri‐implant diseases requires a comprehensive understanding of the etiology and risk factors of these diseases (Kissa et al., 2021). A model that predicts potential peri‐implant disease and implant failure can provide clinicians and patients with information to make informed decisions about modifying risk factors or choosing an alternative treatment.

Statistics on prevalence of the peri‐implant diseases in Syria are unavailable; this research is considered the first of its kind that assesses the condition of the tissues surrounding the implants, specifies the rate of prevalence of the peri‐implant diseases, and assesses the probable factors of risk within the Syrian community's university environment.

## 2 MATERIALS AND METHODS

### Study design and ethics statement

A cross‐sectional study to assess the prevalence and factors of risk of peri‐implant diseases for the patients who received the implanting treatment between 2015 and 2021. The study's community consists of individuals who were treated by many postgraduate students under the supervision of several members of the teaching staff at Damascus University's Periodontology Department. The research was conducted according to the (STROBE) guidelines (Wada et al., 2021) and the Declaration of Helsinki, and the research protocol was ethically approved by the Local Research Ethics Committee of the Faculty of Dentistry at Damascus University (UDDS‐22042021/SRC‐545).

### 2.1 Patient sample

Information about study participants was collected from the department database and they were contacted, but contact was unsuccessful with 49 patients. Out of 186 patients called, 142 patients were referred and asked to the department's clinic for clinical examination and radiological. Three hundred and eighty implants were placed for these 142 patients. All the patients were educated about this study's aims and the wanted clinical and radiographic measures to take and their consent was obtained. Clinical and radiographic examination was performed between March and September 2022.

### 2.2 Sample size calculation

The sample size was computed using the G‐Power v.3.1 software (Universität Kiel), based on variables and outcomes from a prior study (Daubert et al., 2015). The effect size is therefore (0.2). The value of (a) is the first type error (0.3) and the value of (b) is the second type error (0.69).

### 2.3 Inclusion and exclusion criteria

The standards of inclusion were as follows: The patients who received treatment by one implant or more between 2015 and 2021 at the Periodontology Department, possess a file that includes the implanting data at the department along with a functional loading for 12 months at least and accepted the invitation by telephone for their implants to be assessed.

Exclusion criteria were as follows: Patient received phone calls but refused to participate in the study; and dental implants with a functional load duration of less than 12 months.

### 2.4 Data collecting

The participants who agreed to take part in this study underwent a data‐collecting process: demographic data collection and medical/dental history, clinical and radiographic examination.

The following study variables were assessed:

The patient: gender (male or female), age in years, smoking (nonsmoker or smoker), alcohol consumption (yes/no), educational level (primary school/middle grade/high school/university/college), diabetes (yes/no), and such information was reported by the patients. The existing teeth were examined, and periodontal status (periodontitis/healthy).

### 2.5 Clinical and radiographic assessment

Clinical evaluation was carried out by a single investigator and was previously calibrated using a manual periodontal probe (PCP‐UNC15: Hu‐Friedy).

The following peri‐implant clinical parameters were recorded:

Probing depth (PD) in six sites per implant (mesiobuccal, buccal, distobuccal, mesiolingual, lingual, distolingual).

**Figure 1:**
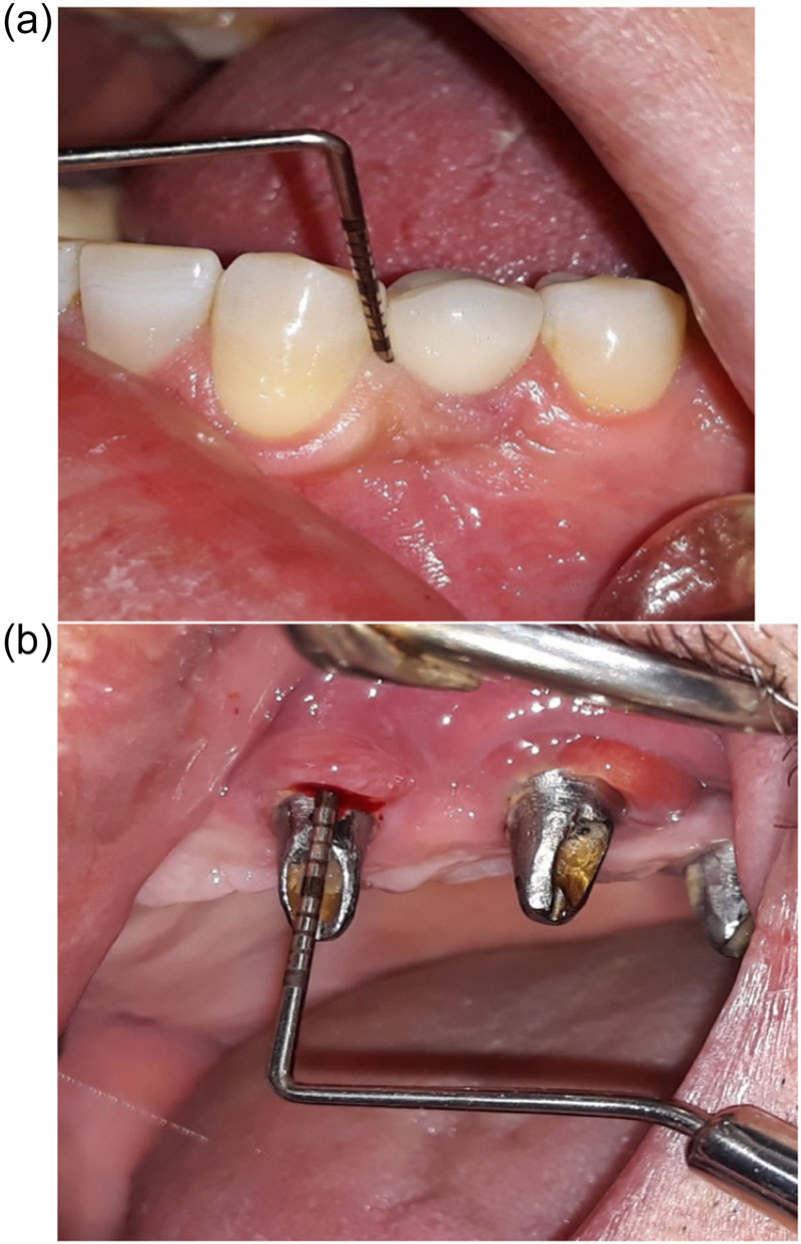
Clinical assessment. (a) Probing depth (PD) and (b) bleeding on probing (BOP).

The PD was defined as the distance from the margin of the mucosal tissue around the implant to the base of the pocket when a slight force was applied (Figure 1a), plaque index (PI) (Arunyanak et al., 2019), bleeding on probing (BOP), and suppuration (SUP) were measured by presence/absence at six sites per implant (BOP/SUP, within 30 s) (Figure 1b).

**Figure 2:**
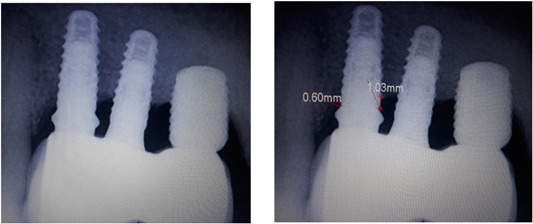
Radiographic assessment: The radiographic bone level is defined as the distance from the implant platform to the first implant/bone contact at the mesial and distal aspects of the implant.

The periapical radiograph was taken with the digital sensor (RVG SYSTEM) of the implant at the time of follow‐up examination using the sensor holder. Bone levels were measured using a digital radiographic display system “Deep View® Software.” The radiographic bone level is defined as the distance from the implant platform to the first implant/bone contact at the mesial and distal aspects of the implant and was measured in mm by a calibrated examiner. The most elevated measurement (mesial or distal) was selected to represent (Kissa et al., 2021) (Figure 2).

### 2.6 Investigator calibration

Clinical examination was performed by two examiners (two PhD students in the Department of Periodontology). Calibration was performed to assess inter‐examiner accuracy. The first five patients were both supplemented to assess internal rater reliability for clinical measures of PD, PI, and BOP; and periodontal condition. The two examiners independently evaluated all radiographs to measure the degree of bone loss in the proximal surfaces of each implant to reach an agreement on the state of the peri‐implant. If there is a difference of opinion, both examiners together take an additional measurement to try to reach an agreement. If they are unable to reach an agreement, the radiographs will be reexamined with a third examiner (a professor in the Department of Periodontology) to reach a consensus on the peri‐implant condition. In this calibration method, we relied on a previous study (Daubert et al., 2015).

### 2.7 Case definition

The following case definitions were considered for the analyses of the study.

Patients with periodontitis were defined as the existence of periodontal PD ≥4 mm with BOP and clinical attachment loss >2 mm in at least two nonadjacent interproximal areas (Papapanou et al., 2018).

Peri‐implant mucositis was defined as the presence of BOP without PD ≥6 mm and a bone level ≥3 mm apical to the most coronal portion of the intraosseous part of the implant.

Peri‐implantitis was defined as the presence of PD ≥6 mm with BOP or SUP and radiographic signs of a bone level ≥3 mm apical to the most coronal portion of the intraosseous part of the implant, this definition has been used in recent studies (Kissa et al., 2021; Windael et al., 2021). The remaining cases are considered peri‐implant health.

Initial radiographs were not available but the case definitions were considered appropriate for this problem. Epidemiological studies should perfectly include previous examinations performed after the first year of functional loading. In the lack of previous radiographic examinations, bone levels ≥3 mm apical of the most coronal portion of the intra‐osseous part of the implant jointly with BOP are reliable with the diagnosis of peri‐implantitis (Berglundh et al., 2018).

Concerning assessment of the prevalence rate at the patient's level, a single patient may be listed under several categories at the same time if the patient has several implants and each implant with a peri‐implant condition differs from his other implants.

### 2.8 Statistical analysis

The data was analyzed using the statistical software (SPSS, Inc.) (SPSS 25.0) and statistical tests were performed at 0.05 level at significance where the following analytical statistical tests were employed.

Shapiro−Wilk test showed a normal distribution of the data.

Descriptive analysis of study data was made to describe the sample and its characteristics, to determine prevalence, and to know the percentage.

Multivariate analytical model was used to study variables and their relationship with the state of tissues surrounding the implant to determine which variable serves as a risk factor in disease identification around the implant.

## 3 RESULTS

### 3.1 Descriptive statistics of the study population

The present analysis included a total of 142 patients with a mean age at examination of (50.9 ± 12.49) years, the study involved 380 dental implants. Table 1 provides a descriptive analysis of the demographic variables of the research sample and patient data.

**Table 1:** Descriptive analysis of the demographic variables of the research sample and patient data.

		*N*	Percentage
Gender	Female	65	45.7%
	Male	77	54.3%
Educational level	Primary school	35	24.6%
	Middle grade	28	19.7%
	High school	42	29.5%
	University/college	37	26.0%
Smoking	No	80	56.3%
	Yes	62	43.7%
Alcohol	No	140	98.6%
	Yes	2	1.4%
Diabetes	No	133	93.7%
	Yes	9	6.3%
Periodontitis	No	58	40.8%
	Yes	84	59.2%

Abbreviation: N, number.

Most of the included patients were males (54.3%), the predominant level of education is high school (29.5%), currently nonsmokers (56.3%), patients with periodontitis (59.2%), and diabetes patients (6.3%). The clinical parameters were reported in (Tables 2 and 3).

**Table 2:** Clinical parameters.

		*N*	Percentage
Plaque index	0	80	21.1%
	1	212	55.8%
	2	75	19.7%
	3	13	3.4%
Suppuration	Absence	371	97.6%
	Presence	9	2.4%
Bleeding on probing	Absence	162	42.6%
	Presence	218	57.4%

Abbreviation: N, number.

**Table 3:** Clinical parameters.

	*N*	Mean	Minimum value	Maximum value	SD
Radiographic bone level	380	1.75	0.10	9.90	1.73
Probing depth	380	4.43	1.00	8.00	1.44

Abbreviations: N, number; SD, standard deviation.

### 3.2 Prevalence of peri‐implant health and diseases

The prevalence of peri‐implant health and diseases is reported in (Table 4). At patient‐level, the prevalence of peri‐implant health was (60.6%) of peri‐implant mucositis of (58.5%) and of peri‐implantitis of (25.4%).

**Table 4:** Prevalence of peri‐implant diseases.

			*N*	Percentage
At patient level	Peri‐implant health	No	56	39.4%
		Yes	86	60.6%
	Peri‐implant mucositis	No	59	41.5%
		Yes	83	58.5%
	Peri‐implantitis	No	106	74.6%
		Yes	36	25.4%

Abbreviation: N, number.

### 3.3 Factors of risk for peri‐implant diseases

According to the multivariate analytical model, only the following factors remained significant at the 0.05 level (Table 5): gender female (peri‐implant mucositis [OR = 0.269; 95% CI: 0.131−0.552] and peri‐implantitis [OR = 0.561; 95% CI: 0.561−0.216]), diabetes (peri‐implant mucositis [OR = 3.4; 95% CI: 1.73−12.73]), periodontitis (peri‐implant mucositis [OR = 2.409; 95% CI: 1.760−2.613], peri‐implantitis [OR = 10.445; 95% CI: 4.097−26.629]).

**Table 5:** Factors of risk associated with peri‐implant diseases: multivariate analytical model at the patient‐level.

		*p* Value	OR	95% CI	
Gender (female)	Health	Ref	Ref	Ref	Ref
	Peri‐implant mucositis	*p* ˂ 0.001^a^	0.269	0.131	0.552
	Peri‐implantitis	0.034^a^	0.561	0.216	1.455
Age	Health	Ref	Ref	Ref	Ref
	Peri‐implant mucositis	0.34	1.012	0.988	1.036
	Peri‐implantitis	0.07	0.973	0.943	1.003
Educational level	Health	Ref	Ref	Ref	Ref
	Peri‐implant mucositis	0.17	1.169	0.936	1.459
	Peri‐implantitis	0.61	0.925	0.684	1.250
Smoking status	Health	Ref	Ref	Ref	Ref
	Peri‐implant mucositis	0.22	1.521	0.780	2.966
	Peri‐implantitis	0.40	0.681	0.280	1.654
Alcohol	Health	Ref	Ref	Ref	Ref
	Peri‐implant mucositis	1.00	1.01	1.633	1.635
	Peri‐implantitis	1.00	1.475	0.000	0.000
Diabetes (yes)	Health	Ref	Ref	Ref	Ref
	Peri‐implant mucositis	0.031^a^	3.4	1.73	12.73
	Peri‐implantitis	1.00	2.64	3.41	10.23
Periodontitis	Health	Ref	Ref	Ref	Ref
	Peri‐implant mucositis	0.026^a^	2.409	0.760	2.613
	Peri‐implantitis	*p* ˂ 0.001^a^	10.445	4.097	26.629

Abbreviations: CI, confidence interval; OR, odds ratio; Ref, reference.

^a^ Statistically significant.

## 4 DISCUSSION

### 4.1 The results of peri‐implant diseases prevalence

This cross‐sectional study showed that peri‐implant diseases are common in patients who received treatment with dental implants in Syria where 58.5% of the patients were diagnosed with peri‐implant mucositis and 25.4% with peri‐implantitis.

The current analysis found an increased prevalence of peri‐implant mucositis in comparison with some other studies. In the previous cross‐sectional studies, the percentage of patients with unhealthy implants was 53.3%−70% where (35%−38.3%) of such patients were diagnosed as peri‐implant mucositis and (14.5%−35%) were diagnosed as peri‐implantitis (14.5%−35%) (Derks et al., 2016b; Vignoletti et al., 2019).

The results of the present analysis may be related to the characteristics of the study population of Syrian patients; and another important factor that may explain the difference in the diagnostic criteria used to define peri‐implant disease (Renvert et al., 2018). A systematic review reveals that there is currently a lack of a uniform diagnostic and classification method for peri‐implantitis. Therefore, based on the present evidence, the rationale for the diagnosis of peri‐implantitis is suggested (Ramanauskaite & Juodzbalys, 2016).

### 4.2 The peri‐implant disease factors of risk

This current study could mention several factors associated with peri‐implant disease since we employed a multivariate analytical model to assess the connections of the variables relating to the patient along with the results of the assessment of the condition of the tissues surrounding the implants.

Patient‐reported information has been recognized for decades as a means of capturing subjective views as well as informing and improving clinical practice. One such measure is self‐reported health (Ahmed et al., 2012). A single question about self‐reported health status may be an appropriate metric for health professionals to determine subjective well‐being and reveal the need to target lifestyle factors in healthy individuals or with disease (Jepsen et al., 2014).

Hormonal effects, such as pregnancy or postmenopausal women, may affect the periodontal tissues. Clinical studies in rodents and larger animals have revealed the negative impact of oophorectomy, a model of postmenopausal hypogonadism, on the structural integrity of the jaw. Because the jawbone is the fulcrum for teeth and dental implants, postmenopausal women are considered at risk for tooth loss and implant loss (Dvorak et al., 2011). This study observed while assessing the risk variables at the patient's level that females constitute a factor of risk compared to males as to the peri‐implant mucositis (OR = 0.269) and the peri‐implantitis (OR = 0.561). Most probably this result is due to the hormonal effect which has been guided for our explanation by the Papalou et al. (2022). Study since there has been an obvious increase in the PD and the BOP in women as such results are explained by the hormonal effect. The results of this current analysis do not agree with the Matarazzo et al. (2018). Study which observed that peri‐implantitis has been largely associated with males, while the effect of gender on the condition of the tissues surrounding the implants was not observed that much in other studies (Jemt et al., 2017).

Diabetes is a nominal variable with only two possible values. Thus, we want to know the number (frequency) of patients with diabetes and what proportion of the total sample they represent as in a previous study (Kissa et al., 2021). Based on the results of the multivariate analytical model, this study found that diabetes increases the risk of occurrence of peri‐implant mucositis (3.4) times as such results differ from Monje et al. (2017). Study who found that the diabetes patients were subjected to the risk of having peri‐implantitis but not peri‐implant mucositis, while the Gunpinar et al. (2020). Study's multivariate analysis did not detect a connection to development of the peri‐implant disease. Whether there is or there is no diabetes has been reported by the patient's report since diabetes patient's report was regarded as a suitable tool for clinical practice (Bowlin et al., 1996). However, the question of how people can understand and explain the concept of self‐reported health conditions remains empirically imperfect (Jepsen et al., 2014). This may demonstrate the difference in results between studies.

Periodontitis its main effect as a factor of risk on the peri‐implantitis lies in that it increases the risk of occurrence (10.4) times. The present study does not agree with some literature (Rokn et al., 2017; Schwarz et al., 2015), which has not proven an increasing risk of having peri‐implantitis in patients who have a history of periodontitis. High tooth and implant survival ratios were observed in patients with periodontitis registered SPC after a median follow‐up of at least 10 years. Moreover, the presence of one or two adjacent teeth does not seem to have any effect on the change in the peri‐implant bone level (Weigel et al., 2023). There is a controversy in the literature about the probable relationship between periodontitis and peri‐implantitis. Such controversy concentrates on the findings of the different studies due to the variation in definitions of periodontitis and peri‐implantitis conditions. There is also another possible reason that we in our study assessed the periodontic condition at the time of the clinical examination while other studies assessed the periodontic condition at the time of implanting. Patients requiring rehabilitation with dental implants should be adequately informed before treatment that the risk of implant failure increases significantly with a history of periodontitis and the absence of SPC (Roccuzzo et al., 2022).

The results of this cross‐sectional study may be affected by several limitations; first, the cross‐sectional design of our study cannot explain causality simply because it considers a single assessment period rather than providing information on disease development. Second, the study's sample has been of one university group which cannot be thus regarded as a random sample; Therefore, multicenter studies with a larger number of randomly selected patients are suggested in future studies. Third, Clinical parameters were evaluated while keeping the prosthesis in place. Therefore, the converging abutment profile as well as the design of the prostheses can affect the accuracy of the PD values. Fourth, In the current study of medical records, smoking was a categorical variable, with two groups, since each participant can be categorized only as either a nonsmoker or a smoker, as in previous studies (Matarazzo et al., 2018; Obreja et al., 2021), but patients who had quit smoking were accepted as nonsmokers and taking into account only their current smoking status. Thus, this may have masked the true impact of smoking on the development of peri‐implant disease; Therefore, it is suggested to study the smoking factor (former smoker to current smoker) and also to include the number of cigarettes per day. In addition, some of the potential risk factors examined were not collected using gold‐standard methods (e.g., self‐assessment history). Because the sample size calculation is based on diffusion data, some of the risk factors tested may not have the statistical power to be included in the final model.

## 5 CONCLUSIONS

The peri‐implant diseases are common in a university sample of Syrian communities and many patient‐related variables (female gender, diabetes, periodontitis) are associated with the progress of peri‐implant diseases.

## AUTHOR CONTRIBUTIONS

Asmaa A. A. Aljalloud conceptualized the idea, provided the clinical and radiographical investigation, and contributed to the writing and documenting. Suleiman Dayoub conceptualized the idea and supervised the MSc thesis for Asmaa A. A. Aljalloud. Yasser Alsayed Tolibah contributed to the interpretation of data and the revision, formatting, and reediting of the manuscript. All authors read and agreed to the published version of the manuscript.

## CONFLICT OF INTEREST STATEMENT

The authors declare no conflicts of interest.

## ETHICS STATEMENT

The study was conducted according to the guidelines of the Declaration of Helsinki and approved by the Local Research Ethics Committee of the Faculty of Dentistry, Damascus University (UDDS‐22042021/SRC‐545). Informed consent was obtained from all subjects/caregivers involved in the study.

## Data Availability

Deidentified data are available upon written request to the corresponding author.
